# Changes in body composition in patients with malignant pleural mesothelioma and the relationship with activity levels and dietary intake

**DOI:** 10.1038/s41430-021-01062-6

**Published:** 2022-01-17

**Authors:** Emily Jeffery, Y. C. Gary Lee, Robert U. Newton, Philippa Lyons-Wall, Joanne McVeigh, Deirdre B. Fitzgerald, Leon Straker, Carolyn J. Peddle-McIntyre

**Affiliations:** 1 Exercise Medicine Research Institute, Edith Cowan University, Joondalup, WA Australia; 2 School of Medical and Health Sciences, Edith Cowan University, Joondalup, WA Australia; 3 School of Population Health, Curtin University, Perth, WA Australia; 4 Respiratory Department, Sir Charles Gairdner Hospital, Nedlands, WA Australia; 5 Institute for Respiratory Health, Nedlands, WA Australia; 6 Medical School, University of Western Australia, Crawley, WA Australia; 7 School of Allied Health, Curtin University, Perth, WA Australia; 8 Movement Physiology Laboratory, School of Physiology, University of Witwatersrand, Johannesburg, South Africa

**Keywords:** Malnutrition, Lung cancer

## Abstract

**Background:**

Skeletal muscle loss is common in advanced cancer and is associated with negative outcomes. In malignant pleural mesothelioma (MPM), no study has reported body composition changes or factors associated with these changes. This study aimed to describe changes in body composition over time and its relationship with activity levels, dietary intake and survival.

**Methods:**

The study was a secondary analysis of data collected from a longitudinal observational study of patients with MPM. Participants completed 3-month assessments for up to 18 months. Participants with two dual-energy x-ray absorptiometry (DXA) scans were included. Changes in appendicular skeletal muscle mass (ASM) and total fat mass were used to categorise participants into phenotypes. Activity levels were measured with an ActiGraph GT3X+ accelerometer and energy and protein intake was measured with a 3-day food record and 24-h recall.

**Results:**

Eighteen participants were included (89% men, mean age 68.9 ± 7.1 years). Median time between DXA was 91 [IQR 84–118] days. Compared to participants with ASM maintenance (*n* = 9), fewer participants with ASM loss (*n* = 9) survived ≥12 months from follow-up (*p* = 0.002). Participants with ASM loss increased sedentary time (*p* = 0.028) and decreased light activity (*p* = 0.028) and step count (*p* = 0.008). Activity levels did not change in participants with ASM maintenance (*p* > 0.05). Energy and protein intake did not change in either group (*p* > 0.05).

**Conclusions:**

Muscle loss was associated with poorer survival and decreased activity levels. Interventions that improve physical activity or muscle mass could benefit patients with MPM.

## Introduction

Malignant pleural mesothelioma (MPM) is an incurable cancer that results from asbestos exposure [1]. Patients with MPM have limited treatment options and short median survival of 12 months [1]. It has been hypothesised that cancer cachexia could contribute to the cause of death in MPM [2].

Cancer cachexia is a form of malnutrition characterised by the loss of skeletal muscle mass in the presence or absence of loss of fat mass, and is often accompanied by anorexia and systemic inflammation [3]. Cancer cachexia can lead to the development of low skeletal muscle mass, which is associated with a range of negative outcomes in advanced cancer populations including poorer quality of life [4], lower activity levels [5], increased treatment toxicity [6], and poorer survival [6]. Further, people with both low skeletal muscle mass and excess fat mass (i.e., sarcopenic obese) have had a greater risk of negative outcomes [7].

In our previous research, we reported that 50% of patients with MPM had low skeletal muscle mass close to the time of diagnosis, and of these participants, 11% were obese [5]. While anecdotally clinicians report that patients with MPM become emaciated over the disease course, and often die with a low BMI [2], there is a lack of evidence available on changes in body composition over time to inform the development of interventions to address these concerns.

Physical activity and dietary intake are modifiable factors that could be central to the development of cancer cachexia. Physical activity and dietary protein intake stimulate muscle protein synthesis [8] and in sufficient quantities could protect against the development of low skeletal muscle mass [9, 10]. In addition, lower levels of physical activity and high dietary energy intake can create a positive energy balance resulting in weight gain that is largely an increase in fat mass [11]. At present, there is little research on the relationship between physical activity, dietary intake and changes in body composition in cancer populations. Understanding these relationships could lead to more targeted interventions to prevent and treat cancer cachexia. Therefore, this study in patients with MPM aimed to describe the changes in body composition over time and the relationship between body composition changes and activity levels and dietary intake. A secondary aim of the study was to explore the association between body composition changes and participant characteristics, such as survival.

## Methods

### Study design and participants

The study was a secondary analysis of data collected from a longitudinal observational study that aimed to describe the functional and nutritional status of patients with MPM. Participants were recruited from a pleural disease clinic in Perth, Western Australia. Patients were eligible if they had cytological or histological confirmation of MPM. Patients were excluded if they were aged <18 years, pregnant or lactating, unable to read and understand English, unable to comply with the protocol or participating in an intervention study likely to influence body composition. Participant consent and physician approval were required for participation in the study. Participants completed assessments of body composition, activity levels and dietary intake during routine hospital visits, approximately every 3 months and were followed until death or for a maximum of 18 months. Participants that did not complete body composition scans at two consecutive assessments were excluded from this analysis. The study was approved by the Sir Charles Gairdner Group and Edith Cowan University Human Research Ethics Committees (ID: 2014-124 and 13255).

### Measurements

#### Demographic and medical variables

Demographic and medical data were obtained from participant medical records. Disease progression at follow-up was determined by clinician examination of the computed tomography scan completed closest to the second body composition scan. The Eastern Cooperative Oncology Group (ECOG) performance status was recorded on the date of assessment [12].

#### Anthropometric measures

Weight and height, measured with participants dressed in light clothing with shoes removed, were used to calculate the BMI. Participants were classified as underweight, normal weight, overweight or obese based on World Health Organisation BMI criteria [13].

#### Body composition

Body composition was assessed using whole-body dual-energy x-ray absorptiometry (DXA) scans (Hologic Horizon A, Hologic Inc., Marlborough, MA, USA). DXA is considered a precise measure of body composition, with coefficients of variation reported to be <0.5% for lean mass, and <1.0% for fat mass [14]. Device calibration was completed daily using the Hologic Spine Phantom [15]. Participants wore light clothing with shoes removed, and were asked to remove all metal objects (i.e., glasses, jewellery). Participants were positioned in a supine position in the centre of the table, their arms by their side and palms facing down, with legs were shoulder-width apart and internally rotated [16]. Participants who were unable to lie flat were given a pillow to support their head [15]. Following the scan, measured weight was compared to total mass to check for any discrepancies. DXA scans were analysed using the in-built scan analysis software (version 13.5.2). To analyse appendicular skeletal muscle and fat mass, a single researcher (EJ) manually corrected the separation of body regions, so that the arms were separated at the acromio-humeral joints and the legs were separated at the pelvic-femoral joints [17]. Low skeletal muscle mass was defined as BMI-adjusted appendicular skeletal muscle mass of 0.86 kg/kg/m^2^ for men and 0.59 kg/kg/m^2^ for women [18]. The cut-points for appendicular skeletal muscle mass were derived from Hologic DXA devices and set as two standard deviations below the mean of a reference sample of young Australian adults [18]. Participants with low skeletal muscle mass were categorised as having low muscularity [19]. Change in body composition variables was calculated as the per cent change between the second and first measurements.

To characterise changes in body composition over time, participants were categorised into body composition phenotypes according to changes in skeletal muscle mass and fat mass. The total lean mass measured with DXA includes both skeletal muscle and residual mass (i.e., organs); however, appendicular lean mass is predominantly skeletal muscle [20]. Therefore, to report on changes in skeletal muscle mass we used appendicular lean mass, known as appendicular skeletal muscle mass, which represents on average, 75% of whole-body skeletal muscle [20]. The four body composition phenotypes were: (1) loss of appendicular skeletal muscle mass and loss of total fat mass; (2) loss of appendicular skeletal muscle mass and maintenance or gain of total fat mass; (3) maintenance or gain of appendicular skeletal muscle mass and loss of total fat mass and (4) maintenance or gain of appendicular skeletal muscle mass and maintenance or gain of total fat mass. A loss was defined as a change of ≥–1.45% for appendicular skeletal muscle mass and ≥–2.15% for total fat mass between the first and second measurements; maintenance or gain was defined as a change of <–1.45% for appendicular skeletal muscle mass and <–2.15% for total fat mass between the first and second measurements. These cut-points represent the minimal detectable change values reported for total lean and fat mass in a study of Hologic DXA devices [14].

#### Activity levels

Sedentary behaviour and physical activity were device-derived following each body composition scan using the ActiGraph GT3X+ accelerometer (Actigraph, Pensacola, FL, USA). The ActiGraph GT3X+ accelerometer has been shown to have high intra- and inter-device reliability, with coefficients of variation ≤2.5% and an intraclass coefficient of 0.99 [21]. Participants were instructed to wear the accelerometer on their hip 24 h/day for 3 days, to only remove for bathing or swimming and to record any non-wear time in a logbook. Cut-points were applied to classify sedentary behaviour as <100 counts/minute (cpm), light activity as 100–1952 cpm and moderate and vigorous physical activity (MVPA) as >1952 cpm [22, 23]. Variables were calculated per day and then averaged across all valid (at least 10 h of data) days. Additional accelerometer methodology for this study has been reported previously [5].

#### Dietary intake

Dietary intake was measured following each body composition scan using a 3-day estimated food record at the initial assessment and a 24-h recall at subsequent assessments. These dietary assessment methods are reported to accurately measure energy and protein intake [24, 25]. Dietary intake data were collected, verified and analysed by a dietitian with experience in dietary intake assessment. To assist participants with the completion of the food record, written and verbal instructions were provided explaining how to complete the food record and estimate portion sizes using household measures. Returned food records were visually inspected and incomplete details were clarified with participants. Participants completed the 24-h recall in a face-to-face interview with a dietitian. 24-h recalls were conducted per the multiple pass method [25]. The food records and 24-h recalls were analysed using Foodworks 8 software (Xyris Software Pty Ltd, Queensland, Australia). Intake variables were calculated per day, and for the food records, intake was averaged across all days. Energy and protein intake were expressed as kJ or g per kg of body weight per day.

### Statistical analyses

Statistical analyses were conducted using the Statistical Package for the Social Sciences (v. 23, IBM Corporation, Somers, NY, USA). Data were expressed as mean (SD) or median [IQR] where the data were not normally distributed. To examine the relationship between changes in body composition and participant characteristics, activity levels and dietary intake, participants were condensed into two groups: (1) participants with a change of ≥–1.45% in appendicular skeletal muscle mass, defined as the muscle loss group; and (2) participants with a change of <–1.45% in appendicular skeletal muscle mass, defined as the muscle maintenance group. Fisher’s exact test was used to test for differences in characteristics between participants with muscle loss and muscle maintenance where the data were categorical. As the data were not normally distributed, the Mann–Whitney test was used to test for differences in characteristics between participants with muscle loss and muscle maintenance where the data were continuous, and for differences in change in activity levels and dietary intake between muscle groups. The Wilcoxon signed-rank test was used to test for differences in body composition, activity levels and dietary intake between the first and second measurements.

## Results

### Participant characteristics

**Fig. 1:**
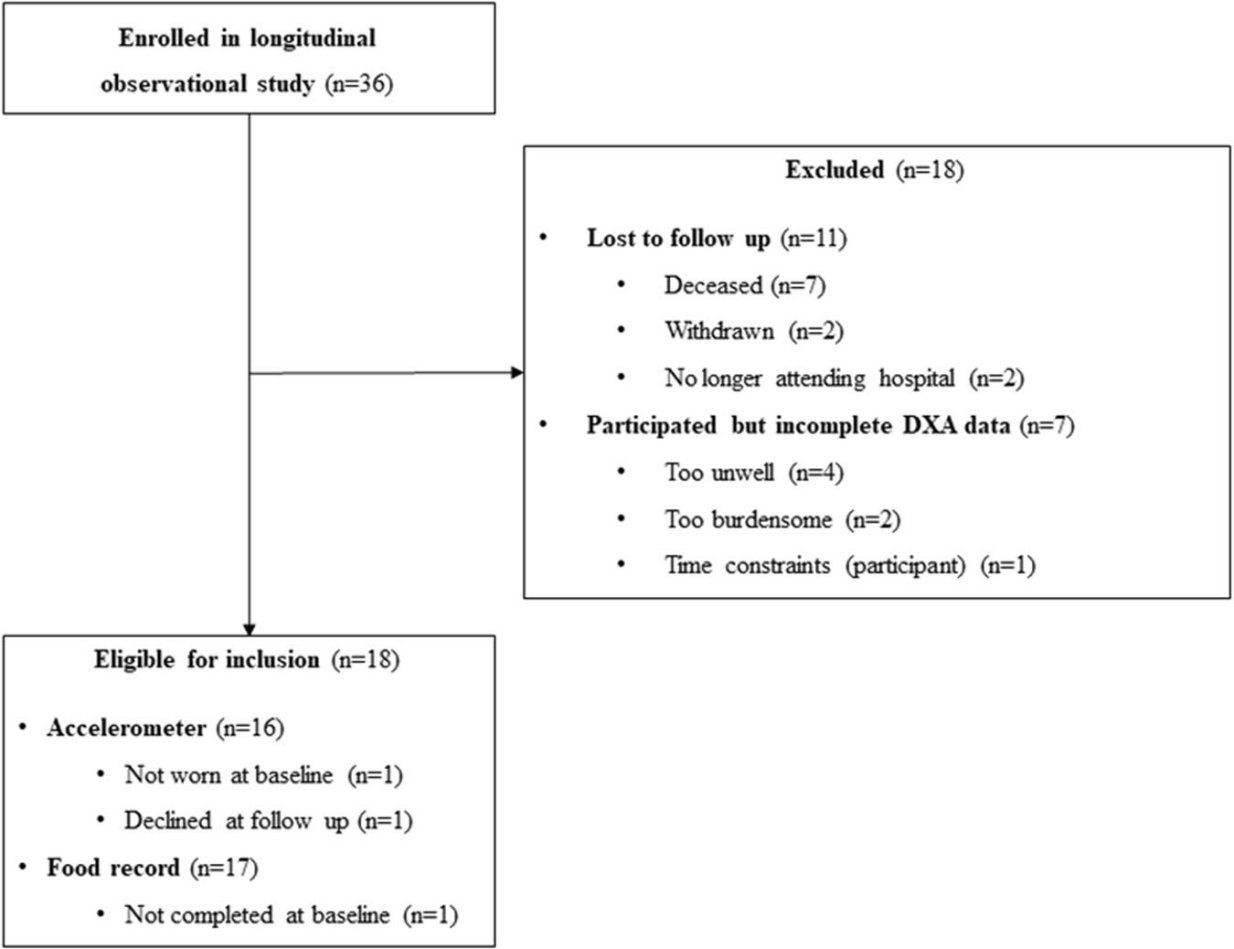
Study flowchart. Participants included and excluded in the study.

Of the 36 patients recruited to the longitudinal observational study, 18 (50%) were included in this study (Fig. 1). The median time between the first and second body composition scans was 91 [84–118] days. Participant characteristics are presented in Table 1. The majority of participants were men (89%) and the mean age of participants was 68.9 (7.1) years. Nine participants (50%) received cancer treatment during the follow-up period. Nine participants (50%) met the criteria for muscle loss and nine participants (50%) met the criteria for muscle maintenance. Seven participants (78%) with muscle loss survived less than 12 months from the second body composition scan, while none of the participants (0%) with muscle maintenance survived less than 12 months from the second body composition scan (*p* = 0.002). For some outcomes presented (e.g., cancer treatment status) the sample size was too small to complete statistical analysis. No other differences in participant characteristics were observed between muscle change groups (*p* > 0.05) (Table 1).

**Table 1:** Participant characteristics, *n* = 18.

	All participants (*n* = 18)	Muscle loss (*n* = 9)	Muscle maintenance (*n* = 9)	*p* value
*Age, years*	68.9 ± 7.1	65.0 [61.0–74.5]	69.0 [63.0–75.0]	0.605
*Sex, men*	16 (88.9%)	8 (88.9%)	8 (100%)	0.471
*BMI, kg/m*^*2*^	25.2 [23.9–28.7]	26.1 [23.9–29.8]	24.6 [23.8–27.1]	0.258
*BMI category*				
Underweight	0 (0.0%)	0 (0.0%)	0 (0.0%)	–
Normal weight range	9 (50.0%)	3 (33.3%)	6 (66.6%)	
Overweight	7 (38.9%)	4 (44.4%)	3 (33.3%)	
Obese	2 (11.1%)	2 (22.2%)	0 (0.0%)	
*Low muscularity, yes*	11 (61.1%)	6 (66.6%)	5 (55.6%)	1.000
*Histological subtype, epithelioid*	15 (83.3%)	7 (77.8%)	8 (88.9%)	1.000
*ECOG performance status at first scan*^a^*, 0–1*	18 (100%)	9 (100%)	9 (100%)	–
*Time from diagnosis to first scan*^a^				
<3 months	10 (55.6%)	4 (44.4%)	6 (66.6%)	–
3–12 months	5 (27.8%)	3 (33.3%)	2 (22.2%)	
>12 months	3 (16.7%)	2 (22.2%)	1 (11.1%)	
*Time from first to second scan, days*	91.0 [84.0–118.0]	85.0 [81.0–94.0]	98.0 [87.0–140.0]	0.113
*Cancer treatment during follow-up, yes*	9 (50.0%)	4 (44.0%)	5 (55.6%)	1.000
*Type of cancer treatment*				
Cisplatin and pemetrexed	3 (33.3%)	1 (25.0%)	2 (40.0%)	–
Carboplatin and pemetrexed	3 (33.3%)	1 (50.0%)	2 (40.0%)	
Vinorelbine	1 (11.1%)	1 (25.0%)	0 (0.0%)	
Clinical trial—cisplatin, pemetrexed, and durvalumab	2 (22.2%)	1 (25.0%)	1 (20.0%)	
*Disease progression at second scan*^a^				
Progressed	10 (55.6%)	5 (55.6%)	5 (55.6%)	–
Stable	4 (22.2%)	2 (22.2%)	2 (22.2%)	
Response to treatment	2 (11.1%)	0 (0.0%)	2 (22.2%)	
Data not available	2 (11.1%)	2 (22.2%)	0 (0.0%)	
*Time from second scan*^a^ *to death*				
<12 months	7 (38.9%)	7 (77.8%)	0 (0.0%)	0.002
≥12 months	11 (61.1%)	2 (22.2%)	9 (100.0%)	

^a^First or second body composition scan.

### Changes in muscularity status

Ten participants (56%) had low muscularity at the first and second body composition scans. One participant (14%) with normal muscularity at the first measurement had low muscularity at the second scan. One participant (9%) who had low muscularity at the first measurement had normal muscularity at the second scan. Of the participants with low muscularity, two (18%) were obese.

### Changes in body composition

**Fig. 2:**
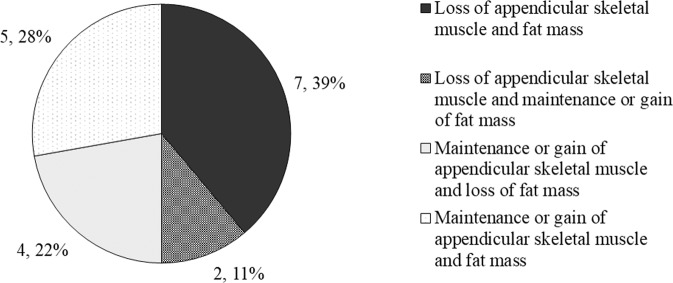
Proportion of participants within each body composition phenotype. (1) Loss of appendicular skeletal muscle mass and loss of total fat mass; (2) loss of appendicular skeletal muscle mass and maintenance or gain of total fat mass; (3) maintenance or gain of appendicular skeletal muscle mass and loss of total fat mass; and (4) maintenance or gain of appendicular skeletal muscle mass and maintenance or gain of total fat mass.

When participants were condensed into the four body composition phenotypes, seven participants (39%) had a loss of appendicular skeletal muscle and fat mass, two participants (11%) had a loss of appendicular skeletal muscle and maintained fat mass, four participants (22%) maintained appendicular skeletal muscle and lost fat mass and, five participants (28%) maintained appendicular skeletal muscle and fat mass (Fig. 2).

There were no significant changes in total, lean or fat mass during the follow-up period in the whole group (Table 2). Participants with muscle loss (*n* = 9) experienced a significant decrease in total mass (*p* = 0.008), trunk lean mass (*p* = 0.011), appendicular skeletal muscle mass (*p* = 0.008) and trunk fat mass (*p* = 0.021) but not appendicular fat mass (*p* = 0.859). Participants with muscle maintenance (*n* = 9) experienced a significant increase in appendicular skeletal muscle mass (*p* = 0.038) but not total mass (*p* = 0.31) trunk lean mass (*p* = 0.594), trunk fat mass (*p* = 0.767) or appendicular fat mass (*p* = 0.594).

**Table 2:** Participant changes in body composition, *n* = 18.

	All participants (*n* = 18)			Muscle loss (*n* = 9)			Muscle maintenance (*n* = 9)		
Mass (kg)	First scan	Second scan	*p* value	First scan	Second scan	*p* value	First scan	Second scan	*p* value
Total	75.0 (70.3–87.4)	74.1 (67.3–88.7)	0.133	75.0 (70.4–91.6)	72.5 (66.9–88.7)	0.008*	74.1 (68.4–87.1)	75.7 (68.8–90.9)	0.314
*Lean mass*									
Total	50.4 (47.3–53.9)	49.5 (46.9–53.9)	0.248	51.4 (46.9–56.4)	48.7 (43.9–54.6)	0.008*	49.6 (46.8–53.5)	50.8 (49.0–53.7)	0.038*
Trunk	26.6 (24.8–29.1)	25.4 (24.5–28.3)	0.085	26.9 (25.2–29.9)	25.2 (23.8–29.0)	0.011*	25.2 (24.7–28.9)	26.5 (24.8–28.2)	0.594
Appendicular	21.4 (18.9–22.2)	20.5 (19.7–22.2)	0.306	21.4 (18.6–23.0)	20.4 (17.3–21.8)	0.008*	21.2 (18.5–22.0)	21.8 (20.3–22.2)	0.011*
*Fat mass*									
Total	24.3 (20.1–30.1)	24.2 (19.8–28.7)	0.215	25.9 (20.1–30.1)	24.3 (20.5–28.0)	0.066	22.9 (16.8–30.1)	23.6 (16.2–31.1)	0.953
Trunk	12.3 (9.8–15.1)	11.2 (9.4–15.1)	0.184	12.4 (10.1–15.3)	10.8 (9.4–14.0)	0.021*	11.9 (7.8–15.5)	11.8 (7.7–17.0)	0.767
Appendicular	11.3 (8.3–13.8)	11.5 (8.4–13.9)	0.679	11.9 (8.3–15.2)	11.5 (8.7–14.2)	0.859	10.2 (8.2–13.0)	10.1 (7.5–14.0)	0.594

Data are presented as median (interquartile range).

*p < 0.05.

### Change in activity levels according to muscle change group

There were no significant changes in activity levels during the follow-up period in the whole group (Table 3). Participants with muscle loss had a significant decrease in median step count (*p* = 0.008), an increase in the proportion of waking hours spent as sedentary (*p* = 0.028) and a decrease in the proportion of waking hours spent in light activity (*p* = 0.028) (Table 3). There was no significant change in the proportion of waking hours spent in MVPA (*p* = 0.260). Participants with muscle maintenance maintained step count (*p* = 0.176), the proportion of waking hours spent as sedentary (*p* = 0.499), in light activity (*p* = 0.499) and in MVPA (*p* = 0.176) (Table 3).

**Table 3:** Participant activity levels and dietary intake, *n* = 17.

	All participants (*n* = 17)			Muscle loss (*n* = 9)			Muscle maintenance (*n* = 8)		
	First scan	Second scan	*p* value	First scan	Second scan	*p* value	First scan	Second scan	*p* value
*Activity behaviours*									
Steps, *n*	5505 (4603–6404)	4736 (3608–6843)	0.196	6013 (4111–9117)	4251 (1738–5372)	0.008*	5039 (4582–5653)	5590 (4196–7404)	0.176
Sedentary behaviour, %	70.3 (61.7–73.1)	73.1 (64.0–76.0)	0.196	67.0 (58.1–72.7)	73.7 (66.8–84.2)	0.028*	72.6 (62.8–73.2)	73.1 (63.4–75.7)	0.499
Light activity, %	27.5 (26.4–35.0)	25.5 (22.3–34.5)	0.215	28.4 (26.0–39.6)	25.3 (15.4–30.8)	0.028*	27.1 (26.3–35.1)	26.2 (22.7–35.7)	0.499
MVPA, %	0.8 (0.5–3.1)	0.9 (0.7–1.7)	0.836	1.0 (0.4–5.3)	0.7 (0.3–1.5)	0.260	0.6 (0.5–1.4)	1.7 (0.8–1.7)	0.398
*Dietary intake*									
Energy intake, kJ/kg	129.6 (90.1–143.8)	121.8 (102.3–147.2)	0.981	122.0 (83.2–138.6)	116.2 (81.7–127.4)	0.314	136.8 (103.1–151.1)	142.0 (110.2–245.5)	0.263
Protein intake, g/kg	1.5 (0.9–2.0)	1.4 (1.1–1.8)	0.492	1.0 (0.9–1.9)	1.2 (0.7–1.4)	0.260	1.5 (1.0–2.0)	1.7 (1.4–2.7)	0.069

Data are presented as median (interquartile range).

*MVPA* moderate and vigorous physical activity.

*p < 0.05.

**Fig. 3:**
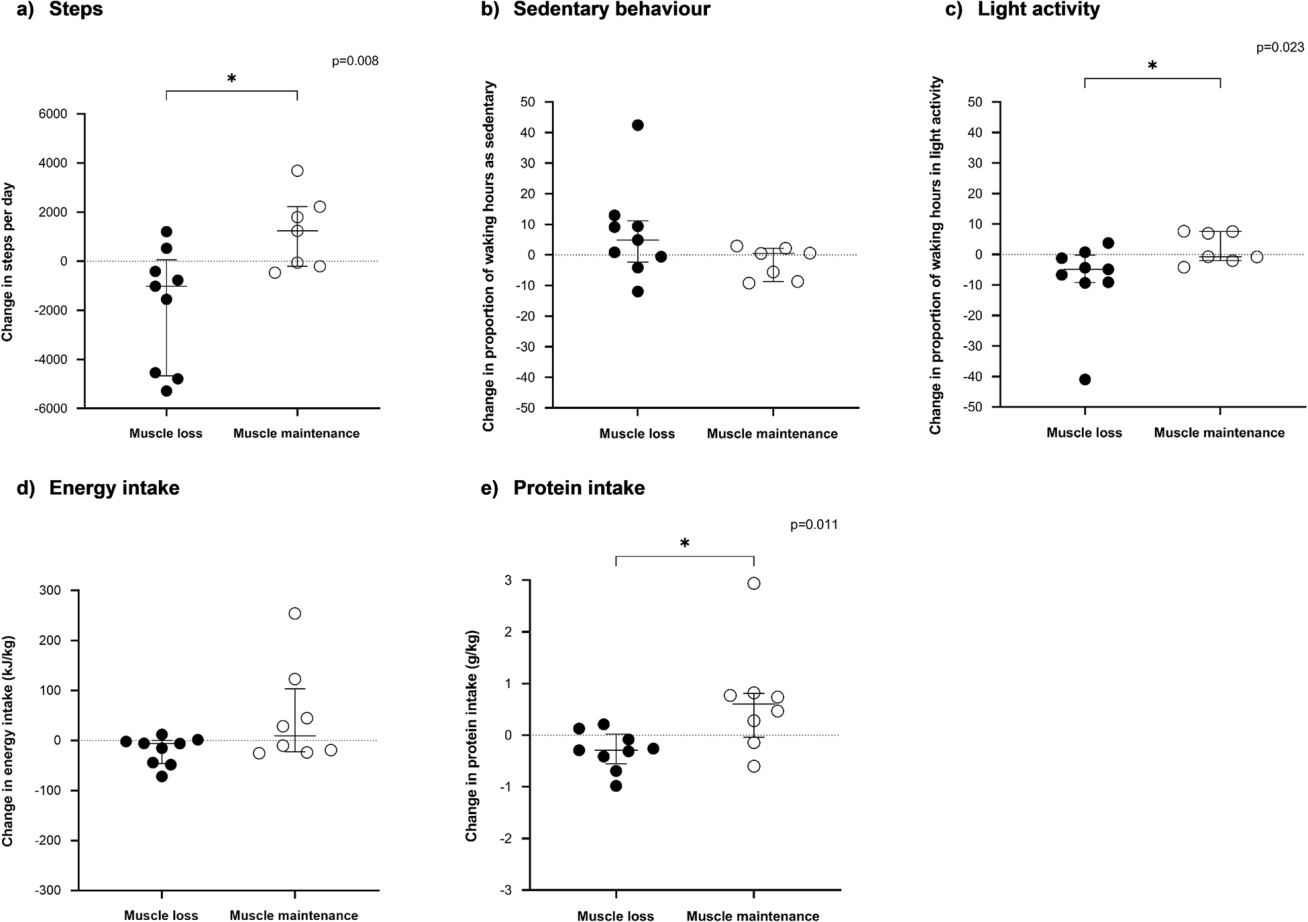
Change in physical activity and dietary intake according to muscle change group. **a** Step count; **b** proportion of waking hours as sedentary; **c** proportion of waking hours in light activity; **d** energy intake (kJ/kg); and **e** protein intake (g/kg). *p < 0.05.

There was a significant difference between participants with muscle loss and muscle maintenance for change in step count (–1020 [–4667 to 56] vs. 1234 [–204 to 2221] steps/day; *p* = 0.008; Fig. 3) and for the proportion of waking hours spent in light activity (–4.8 [–9.2 to 0.2] vs. –0.7 [–2.0 to 7.5]; *p* = 0.023; Fig. 3) but not for the proportion of waking hours spent as sedentary (4.9 [–2.3 to 11.1] vs. 0.5 [–8.6 to 2.2]; *p* = 0.142; Fig. 3).

### Change in dietary intake according to muscle change group

There were no significant within-group changes in energy and protein intake during the follow-up period in the whole group (Table 3). Participants with muscle loss and those with muscle maintenance had no significant change in energy and protein intake (Table 3).

There was a significant difference between participants with muscle loss and muscle maintenance for change in protein intake (–0.29 [–0.55 to 0.02] vs. 0.60 [–0.03 to 0.81] g/kg/day; *p* = 0.011; Fig. 3) but not for change in energy intake (–5.8 [–46.1 to –0.03] vs. 9.4 [–22.5 to 103.6] kJ/kg/day; *p* = 0.236; Fig. 3).

## Discussion

Our study is the first to prospectively assess changes in body composition in relation to activity levels and dietary intake in patients with MPM. We identified multiple patterns of body composition change among our participants. Notably, participants with muscle loss and muscle maintenance had distinct survival, physical activity and dietary intake characteristics.

Our participants could be categorised across all four body composition phenotypes. The most common phenotype, which included 39% of participants, was the loss of appendicular skeletal muscle mass and fat mass, which is consistent with the cachexia phenotype [3]. When we condensed these body composition phenotypes to two groups: (1) muscle loss and (2) muscle maintenance; 50% of participants had muscle loss and 50% had muscle maintenance. This result is particularly notable as the low mean BMI reported in a post-mortem study indicates patients with MPM become emaciated over the disease course [2]. While muscle loss was common, our results suggest that a proportion of participants with MPM can maintain muscle, at least for a proportion of the disease course.

There were significant differences in survival between participants with muscle loss and muscle maintenance. Only a small proportion (22%) of participants with muscle loss survived at least 12 months from the second body composition scan, while all (100%) participants with muscle maintenance survived at least 12 months from the second body composition scan. Therefore, muscle loss could be indicative of shorter survival in patients with MPM. Similar findings have been reported in a large retrospective study of patients with advanced cancer (*n* = 368) [26] where the authors stated that muscle loss became more common as death approached [26]. Tumour burden is thought to mediate the metabolic changes that cause loss of muscle and fat mass [27], highlighting the importance of efficacious cancer treatments for the management of cachexia [3]. There are currently limited treatment options for those with MPM and only 40% of patients respond to first-line chemotherapy treatment [28]. Therefore, addressing lifestyle factors that contribute to cancer cachexia could offer benefits to patients with MPM.

Participants with muscle loss had a significant decline in activity levels over the follow-up period of 3 months, while participants with muscle maintenance maintained their activity levels. As physical activity is required for muscle protein synthesis [8], a decrease in physical activity may have contributed to muscle loss among our participants. In addition, as the majority of participants (70%) with muscle loss had low muscularity at follow-up, participants may not have had the strength and endurance to participate in their usual physical activity. The lack of physical activity could result in an even greater reduction in muscle loss. Therefore, regardless of the causal pathway between muscle loss and activity levels, resistance exercise training may offer benefit to patients with MPM as it can improve skeletal muscle mass, strength and physical function [29].

There were no statistically significant changes in dietary intake over the follow-up period for participants with muscle loss and muscle maintenance; however, we made clinically meaningful observations. Participants with muscle loss had a median energy and protein intake that was within the recommended energy and protein intake range of 105–126 kJ/kg and 1.0–1.5 g/day, respectively [30], while median energy and protein intake among participants with muscle maintenance exceeded these recommendations. A larger study (*n* = 52) in patients with incurable non-small cell lung cancer (NSCLC) reported that higher energy and protein intakes (149 kJ/kg and 1.4 g/kg, respectively) were associated with maintenance of skeletal muscle mass during chemotherapy [31]. Approximately 40–50% of patients with NSCLC are reported to have an elevated resting energy expenditure [32, 33], which could lead to muscle and fat loss unless dietary intake is increased proportionally. As muscle loss developed in our participants meeting dietary intake recommendations, it is possible that elevated resting energy expenditure exists to some extent in patients with MPM. Intakes of energy and protein that exceed recommendations may be needed to preserve skeletal muscle mass in patients with MPM.

This study has several potential limitations worthy of consideration. There are several factors known to affect muscle and fat metabolism, including disease progression, inflammation, cancer treatment and older age [34]. As participants in this study may be at different stages of the disease, combining all participants together could have introduced bias to the findings. While these characteristics were compared between participants with and without muscle loss, the sample size was too small to allow further evaluation concerning changes in body composition. Energy and protein intake at baseline and follow-up were measured using different dietary assessment methods, which could have affected the repeatability of the measurement. Participant feedback indicated that a 3-day food record was burdensome; therefore, we used 24-h recalls at follow-up assessments. Compared with a 24-h recall, a 3-day food record could be more representative of usual dietary intake as the measurement is carried out over a greater number of days; however, a 24-h food recall is not less accurate than a food record [25]. Considering this population of advanced cancer patients, participant burden was a key consideration in our study that should also be taken into account in future investigations.

Our study provides an initial insight into changes in body composition experienced by patients with MPM; however, further research is needed to understand the factors that contribute to the maintenance or decline of physical activity and dietary intake over time. A strength of our study is the use of DXA for body composition analysis, which enabled us to complete a reliable evaluation of appendicular skeletal muscle mass and whole-body and regional fat mass [20]. These data cannot be obtained through computed tomography evaluation of body composition, which employs a single cross-section analysis, and existing prediction equations used to convert cross-sectional data to appendicular skeletal muscle mass may be inaccurate [35]. In addition, we report device-derived sedentary behaviour and physical activity using an accelerometer, which has greater accuracy when compared with self-report questionnaires [36].

## Conclusion

For the first time, we report on body composition changes over time in patients with MPM. Our results indicate that multiple patterns of change in body composition exist in this patient population. Muscle loss was associated with poorer survival and decreased activity levels. Interventions that improve physical activity or muscle mass could benefit patients with MPM.
